# Novel multifunctional triple folic acid, biotin and CD44 targeting pH-sensitive nano-actiniaes for breast cancer combinational therapy

**DOI:** 10.1080/10717544.2019.1669734

**Published:** 2019-10-01

**Authors:** Mengna Liu, Bingjie Wang, Chunjing Guo, Xiaoya Hou, Ziting Cheng, Daquan Chen

**Affiliations:** School of Pharmacy, Yantai University, Yantai, PR China

**Keywords:** Breast cancer and stem cells, triple targeting nanoparticles, pH sensitive, nano-actiniaes, icariin and curcumin co-delivery

## Abstract

In this study, novel multifunctional folic acid, biotin, and CD44 receptors targeted and pH-sensitive “nano-actiniaes” were fabricated with icariin (ICA) and curcumin (Cur) as loaded model drugs for breast cancer therapy. The newly synthesized polymer oligomeric hyaluronic acid-hydrazone bond-folic acid-biotin (Bio-oHA-Hyd-FA) was characterized by ^1^H NMR spectrogram (proton nuclear magnetic resonance). The obtained drug carrier Bio-oHA-Hyd-FA self-assembled into nanomicelles, named as “nano-actiniaes”, in aqueous media with hydrodynamic diameter of 162.7 ± 5 nm. The size, surface zeta potential, and morphology of the “nano-actiniaes” were observed via TEM. The *in vitro* release experiment indicated that much more encapsulated icariin (ICA) and curcumin (Cur) were released from the Bio-oHA-Hyd-FA micelles (nano-actiniaes) in the acidic environment. Additionally, the cytotoxicity research demonstrated that the Bio-oHA-Hyd-FA carrier material was completely nontoxic, and the ICA&Cur “nano-actiniaes” had greater cytotoxicity compared with other control groups. In addition, the “nano-actiniaes” were found to significantly inhibit cancer cell invasion by Transwell assay. Moreover, *in vivo* evaluation of anti-tumor effect illustrated that the ICA and Cur “nano-actiniaes” possessed inhibitory effect on tumors. Consequently, the multi-targeted pH-sensitive “nano-actiniaes” can realize significant tumor targeting and effectively inhibit tumor growth.

## 1. Introduction

An increasing number of researchers have illustrated that tumor growth, drug resistance and development are boosted by a small number of immortalized cells, known as cancer stem cells (CSCs). Consequently, completely eliminating CSCs is critical when treating tumors (Marengo et al., 2019). Breast cancer stem cells (BCSCs) have the ability to self-renew and unlimitedly proliferate, which is the main reason for tumorigenesis, metastasis, and relapse. BCSCs show resistance to anticancer drugs, therefore, this study selected nano-micelles to deliver curcumin and icariin to reduce resistance of BCSCs and breast cancer cells. In addition, many studies have demonstrated that there are multiple specific markers on BCSCs, such as CD24, CD44, and so forth (Ghasemi et al., 2019). These protein markers will make an important impact when targeting BCSCs, and may probably provide a prospective approach to completely cure breast cancer.

Oligomeric hyaluronic acid (oHA) is a macromolecular polysaccharide that has good tumor targeting, biodegradability, non-immunogenicity, nontoxicity, and other properties. As known, hyaluronic acid and its derivatives can bind to specific receptors on external of cancer cells, owing to their high expression, for instance, CD44 receptors, hyaluronan-mediated motility receptor (HMMR) and so on (Cai et al., 2019; Huang and Chen, 2019). According to related reports, CD44 is a receptor on a variety of cell surfaces, and it is overexpressed in diverse solid tumors. Studies such as Muntimadugu et al. reported the use of hyaluronic acid (HA) coated SLM nanoparticles to target tumor cells and their stem cells (Muntimadugu et al., 2016). Moreover, many active groups can be modified in their hyaluronic acid structure. In addition, another critical reason for using hyaluronic acid as a drug carrier is that it can be degraded by multiple enzymes in the tumor microenvironment, allowing anticancer drugs to be released at the site of the disease (Chen et al., 2016; Wang et al., 2018). At present, research on HA has become a hot spot, and its application in drug delivery has received a growing number of attentions.

Folic acid (FA) is an indispensable nutrient for the human body that is composed of pteridine, para-aminobenzoic acid, and L-Glutamic acid. It can enter cells through folate receptors (FRs) (Handali et al., 2018; Zhang et al., 2019). FRs are lowly expressed in normal tissues and highly expressed in breast cancer, ovarian cancer and so on. But beyond that, folic acid has good stability, nontoxicity, easy to modify, and other advantages. These are prerequisites for application of FA as a drug targeting ligand. The use of FA-HA-TOS (TOS: α-tocopherol succinate) conjugates for tumor targeting drug system to deliver the paclitaxel were reported via Zhang et al. (2019). Therefore, drug carriers can be modified with folic acid to strengthen the recognition and internalization by the tumor tissue, thereby improving their targeting efficiency (Ren et al., 2019; Xu et al., 2019). Biotin is a water-soluble micronutrient, also called vitamin H, that is crucial for cell proliferation. For the sake of rapid proliferation and differentiation, tumor cells require greater amount of biotin than normal cells (Rompicharla et al., 2019). Biotin receptors are over-expressed in a variety of malignancies like breast cancer, kidney cancer, and lung cancer, which have a high affinity for biotin. For instance, Kim et al. reported the use of biotin-conjugated PAE-g-PEG-ch micelles for the delivery of doxorubicin to tumor tissue (Kim et al., 2012). A large number of researchers demonstrated that the biotin receptor is high-expressed in those cancer cells where folate receptor is over-expressed (Li et al., 2017; Maiti and Paira, 2018; Zhang et al., 2019). Consequently, biotin has been usually introduced as tumor-targeting ligand to modify polymer-carriers and accumulate anticancer drugs in tumor tissues.

Epimedium is a traditional Chinese herbal medicine, and its main active ingredient is icariin (ICA), which has an anti-tumor effect, such as inhibiting proliferation and differentiation of tumor cells, promoting tumor suppressor gene expression, inducing tumor cell cycle arrest and other effects. In addition, ICA is also used to treat breast lumps and osteoporosis, improve cardiovascular function, increase immune activity and so on. Moreover, studies have indicated that ICA has certain anti-tumor effects on breast cancer, hepatic carcinoma and lung cancer (Jiang et al., 2016; Zhang et al., 2018; Zhou et al., 2019). Icariin itself has anti-tumor activity and can also cooperate with other anti-tumor drugs to treat tumors, such as curcumin, doxorubicin and so on. Curcumin is a chemical component extracted from the rhizome of traditional Chinese medicine turmeric. Clinical study illustrated that curcumin could prevent and treat various cancers such as leukemia, breast cancer, liver cancer, etc. by inhibiting tumor-associated gene expression and angiogenesis (Li et al., 2016; Zhang et al., 2019). Nevertheless, the efficacy of anti-cancer drugs (such as icariin and adriamycin) is reduced because of their low solubility, rapid metabolism, and poor bioavailability. In this study, we used polymeric micelles to co-encapsulate curcumin and icariin to heighten their hydrophilicity and effect.

**Figure 1.**
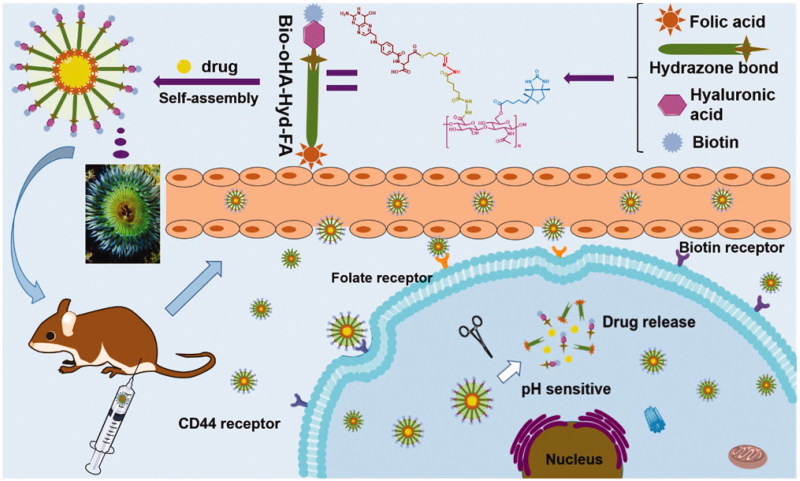
The sketch map of Bio-oHA-Hyd-FA carriers self-assembly into polymeric micelles (“nano-actiniaes”) and the anti-cancer drugs released from nanomicelles in the weakly acidic environment.

In this research, we designed multi-targeting ICA&Cur-loaded micelles based on pH-sensitive hydrazone bond, folic acid and biotin-conjugated hyaluronic acid to effectively deliver curcumin and icariin to tumor tissue, as shown in Figure 1. By making use of folic acid, biotin, and CD44 receptors mediated targeting, the prepared ICA&Cur “nano-actiniaes” enhanced the stability of anti-cancer drugs, with accurate delivery of drugs to tumor tissue and cancer stem cells, thereby ameliorating anticancer efficacy. Moreover, ICA and Cur were released in the microenvironment of the tumor due to their connection to pH-sensitive hydrazone bond (Li et al., 2015). Additionally, the synthesis of Bio-oHA-Hyd-FA copolymer, preparation, and characterization of “nano-actiniaes”, cytotoxicity, transwell invasion assay and anti-tumor effect of “nano-actiniaes” *in vivo* were also examined in detail.

## 2. Materials and methods

### 2.1. Materials

oHA (molecular weight <10 kDa) was purchased from Huaxi Furuida Biomedical Co., Ltd. (Shandong, China). Adipic dihydrazide (ADH) was provided by Tianjin Fuyu Chemical Industrial Corporation (Tianjin, China). Cur and ICA were obtained from Sigma-Aldrich (Shanghai, PR China). N-Hydroxysuccinimide (NHS), 1-Ethyl-3-(3-dimethylaminopropyl) carbodiimide hydrochloride (EDCI) and 4-dimethyl-aminopyridine (DMAP) were provided by Adamas Reagent Co., Ltd. (Shanghai, China). Dimethyl sulfoxide (DMSO), Triethylamine, Glacial acetic acid, and Formamide were obtained from Tianjin Fuyu Fine Chemical Co., Ltd. (Tianjin, China). Folic acid (FA), Biotin, and 3-Acetyl-1-propanol were provided by Aladdin Chemistry Co., Ltd. (Shanghai, China). Deionized water used in the experiment was laboratory-made. MCF-7 cells and breast cancer stem cells (Basal-like) were provided by Shanghai Saiqi Biological Engineering Co., Ltd. (Shanghai, China). Hoechst 33342 was obtained from Sigma-Aldrich. MEM medium, FBS (fetal bovine serum), MTT and 4% paraformaldehyde were acquired from Tianhang Biotechnology Co., Ltd. (Zhejiang, China).

### 2.2. Methods

#### 2.2.1. Synthesis of oligomeric hyaluronic acid and adipic dihydrazide(oHA-ADH)

**Figure 2.**
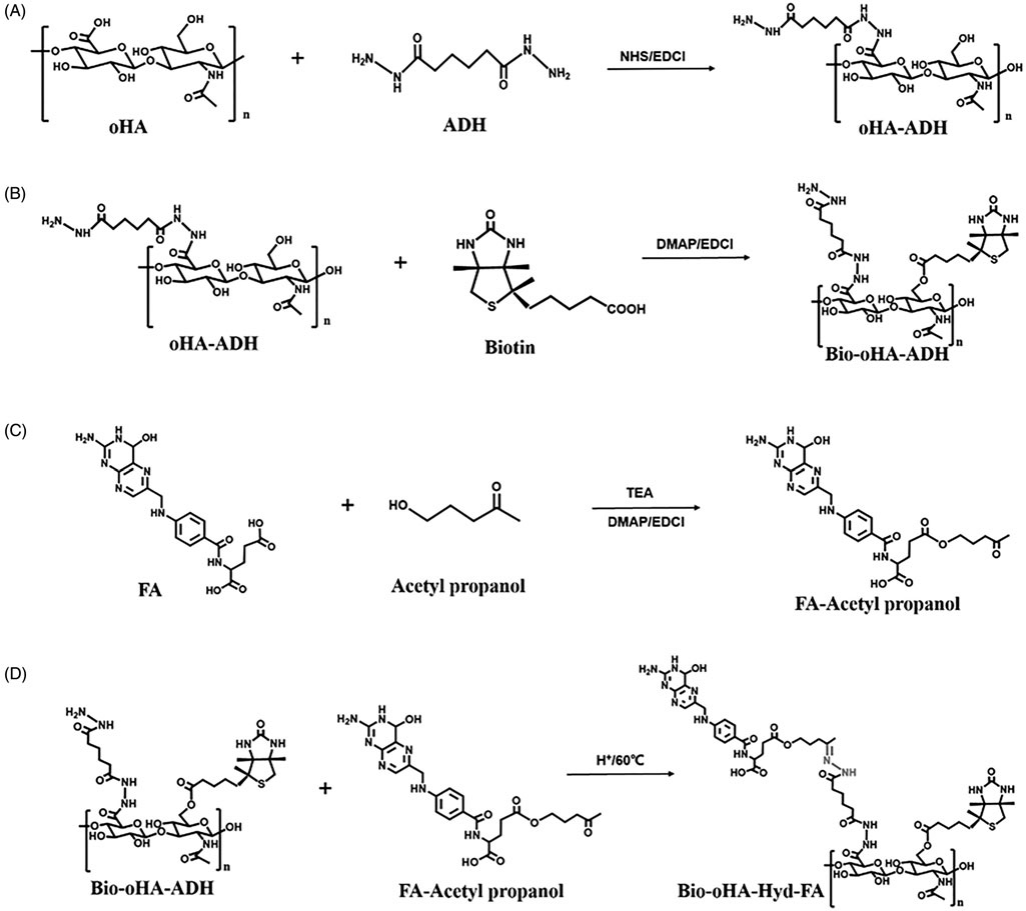
Synthesis scheme of Bio-oHA-Hyd-FA carriers.

As displayed in Figure 2(A), oHA-ADH was synthesized by reacting adipic dihydrazide, oligomeric hyaluronic acid (80.66 mg, 0.2 mM), 1.25 equivalents of EDCI (47.9 mg, 0.25 mM) and 1.1 equivalents of N-Hydroxysuccinimide (42.7 mg, 0.22 mM), which were dispersed in 5 mL of formamide and activated for 3 h at 47 °C. ADH (104.52 mg, 0.6 mM) was dispersed in 3 mL of formamide, followed by subsequent placing in the above mixture, and pH was adjusted to approximately 4.7. After reacting for 20 hours at 55 °C, the pH was adjusted to 7.0, to terminate the reaction. The reacted sample was then separated by dialysis bag at room temperature for 12 h, to remove the side reaction products. The synthetic product oHA-ADH was recovered after freeze-drying for 12 h (Lv et al., 2018).

#### 2.2.2. Synthesis of biotin and oHA-ADH (Bio-oHA-ADH)

As illustrated in Figure 2(B), Bio-oHA-ADH was synthesized via esterification reaction between biotin and oHA-ADH. Briefly, biotin (146.58 mg, 0.6 mM), 0.6 equivalents of DMAP (43.98 mg, 0.36 mM), and 0.7 equivalents of EDCI (80.5 mg, 0.42 mM) were placed in 2.5 mL of formamide. After activation at 41 °C for 5 h, 0.67 equivalents of oHA-ADH (231 mg, 0.4 mM) was dissolved in 4 mL of formamide and mixed with above reactants. The reaction mixture was agitated at 53 °C for 24 h, and the above reactants were dialyzed (MWCO: 2000 Da) with deionized water to clear away unreacted raw materials. Ultimately, the dialyzed mixture was lyophilized to acquire Bio-oHA-ADH.

#### 2.2.3. Synthesis of folic acid, acetyl propanol and Bio-oHA-ADH (Bio-oHA-Hyd-FA)

Folic acid (176.56 mg, 0.4 mM), 0.75 equivalents of DMAP (36.65 mg, 0.3 mM), and 1.25 equivalents of EDCI (95.85 mg, 0.5 mM) were mixed in 3 mL of anhydrous DMSO. Three drops of TEA were then placed into the above mixture at room temperature. After activation at 40 °C for 4 h, 122.5 μL of acetyl propanol (1.2 mM) was mixed with above reactants, and the reaction was protected from light at 57 °C for 12 h. The reactant mixture was dialyzed (MWCO: 300 Da). Then, dialysis product was freeze-dried to obtain folic acid–acetyl propanol.

Next, Bio-oHA-Hyd-FA was prepared by reacting hydrazine group from Bio-oHA-ADH with carbonyl group from folic acid–acetyl propanol. In brief, 287.63 mg of Bio-oHA-ADH (0.35 mM) and 1 equivalents of folic acid- acetyl propanol (195.13 mg, 0.35 mM) were dispersed in 5.5 mL of formamide. After adding 85.8 μL of glacial acetic acid (1.5 mM), the reaction was carried out for 24 h at 60 °C in the dark. The reacted mixture was dialyzed (MWCO: 3000 Da) at room temperature for 12 h to remove unreacted raw materials and by-products. In the end, the Bio-oHA-Hyd-FA conjugates were collected by freeze-drying technique, and the synthesis process is displayed in Figure 2(C,D).

#### 2.2.4. Characterization of Bio-oHA-Hyd-FA

The structure of Bio-oHA-Hyd-FA conjugate was verified by ^1^H-NMR spectroscopy. For the ^1^H-NMR spectra, 6 mg of Bio-oHA-Hyd-FA was then dissolved in the DMSO-D_6_ and D_2_O (3:2) to determine chemical shifts.

#### 2.2.5. Preparation of ICA&Cur “nano-actiniaes”

The ICA&Cur “nano-actiniaes” were obtained by using the membrane dialysis method. In brief, 12 mg of Bio-oHA-Hyd-FA conjugates and an amount of ICA&Cur (at the different mass ratios for drugs) were placed in 5 mL of formamide. The above reactants were then vigorously stirred at 45 °C for 2 h, to completely disperse in formamide. The reactants were dialyzed for 7 h, and the yellow ICA&Cur “nano-actiniaes” solution was acquired by centrifugation. The yellow solution was observed to have obvious Tyndall effect, which confirmed that micelles had been formed.

#### 2.2.6. Characterization of ICA&Cur “nano-actiniaes”

##### 2.2.6.1. Study on particle size, zeta potential and morphology of “nano-actiniaes” by TEM

The average particle size, zeta potential, and polydispersity index (PDI) of ICA&Cur “nano-actiniaes” were detected by using Particle Analyzer Delsa Nano C. The morphological characteristics of “nano-actiniaes” were determined using Transmission Electron Microscopy (TEM, H-600; Hitachi, Japan).

##### 2.2.6.2. Detection of DL% and EE% of ICA&Cur “nano-actiniaes”

DL% and EE% of “nano-actiniaes” were determined using high-performance liquid chromatography (HPLC: Agilent Technologies). In simple terms, 1.5 mL of ICA&Cur “nano-actiniaes” was dispersed in 3 mL methanol, and then placed in a volumetric flask (10 mL) and supplemented to right amount of methanol. The above clear solution was centrifuged into the ultra-centrifugal tube (100,000 Da), and the amount of ICA&Cur in the filtered solution was measured by HPLC with a Phenomenex C18 column. The mobile phase was acetonitrile and 0.5% glacial acetic acid (65:35, v/v) at a flow rate of 1.0 mL/min. The detection wavelengths for Cur and ICA were 425 nm and 270 nm, respectively (Wang et al., 2012; Sang et al., 2018). The drug loading content (DL%) and encapsulation efficiency (EE%) of ICA&Cur “nano-actiniaes” were obtained by the following formulas:

$$\text{EE}\%=\frac{\text{mass}\;\text{of}\;\text{drug}\;\text{in}\;\text{polymeric}\;\text{micelles}}{\text{Mass}\;\text{of}\;\text{the}\;\text{feeding}\;\text{drug}}*100$$

$$\text{DL}\%=\frac{\text{Weight}\;\text{of}\;\text{drug}\;\text{in}\;\text{polymeric}\;\text{micelles}}{\text{total}\;\text{weight}\;\text{of}\;\text{the}\;\text{polymeric}\;\text{micelles}}*100$$

#### 2.2.7. *In vitro* pH-responsive ICA&Cur release research

ICA&Cur were released from the “nano-actiniaes” to assess the pH-sensitive properties of the Bio-oHA-Hyd-FA carrier via dialysis method (Jafarzadeh-Holagh et al., 2018; Wang et al., 2018). Briefly, 7 mL of ICA&Cur “nano-actiniaes” solution was concentrated to 1.5 mL and added into the dialysis bag with a dropper, which was immersed fully in 45 mL PBS with different pH values, and 0.5% Tween-80 was used as a solubilizer of ICA&Cur. The mixture was then placed in water bath and mechanically shaken (100 r/min) at 37 °C. 1 mL of release medium was removed at specified time intervals, and the same amount of fresh release medium was complemented. The amount of ICA&Cur cumulative release from the ICA&Cur “nano-actiniaes” was detected by HPLC.

#### 2.2.8. Cell culture

MCF-7 cells and breast cancer cells were utilized *in vitro* studies of “nano-actiniaes”. These two kinds of cells were cultivated in MEM containing 10% FBS and maintained in a constant temperature environment of 37 °C (Dong et al., 2018).

#### 2.2.9. *In vitro* cell uptake of ICA&Cur “nano-actiniaes”

##### 2.2.9.1. Effect of time on cell uptake

To study cellular uptake of ICA&Cur “nano-actiniaes”, MCF-7 cells and BCSCs were incubated in the 24-well plate (3 × 10^5^ cells/well) at 37 °C, respectively. After culturing the cells for 24 h, the MEM was removed and supplemented with fresh MEM containing free Cur (Cur concentration: 10 μg/mL), free ICA&Cur, Cur-loaded polymeric micelles or ICA&Cur “nano-actiniaes”, followed then by incubation for 0.5 h, 2 h and 4 h in constant temperature incubator. The cells were then fixed with 4% paraformaldehyde solution for 20 min. The cellular uptake result was viewed via fluorescent inverted microscope (Eclipse E400; Nikon Corporation).

##### 2.2.9.2. Concentration-dependent research

After culturing the cells for 30 h, the free Cur, free ICA&Cur, Cur-loaded polymeric micelles, and ICA&Cur-loaded polymeric micelles were substituted for MEM medium, respectively. The MCF-7 cells and BCSCs were incubated for approximately 4 h and fixed with 4% paraformaldehyde. The result was observed using an inverted fluorescence microscope.

#### 2.2.10. Cell cytotoxicity study of Bio-oHA-Hyd-FA and ICA&Cur “nano-actiniaes”

The MTT assay was used to assess the cytotoxicity of free Cur, free ICA, free ICA&Cur, blank Bio-oHA-Hyd-FA, Cur-loaded polymeric micelles, ICA-loaded polymeric micelles and ICA&Cur-loaded polymeric micelles (Chen et al., 2012; Yu et al., 2018). Briefly, the MCF-7 cells and BCSCs were inoculated into 96-well plates (4000 cells/well) and incubated for 24 h in humid environment. About 0.1 mL of different concentrations of free Cur, free ICA, free ICA&Cur, blank Bio-oHA-Hyd-FA, Cur-loaded polymeric micelles, ICA-loaded polymeric micelles, and ICA&Cur-loaded polymeric micelles were diluted with fresh medium after the original MEM was cleared away, and cultivated for 24 h and 48 h. Next, 20 μL of MTT dyeing agent (5 mg/mL) was complemented to every well and incubated for 4 h in humid environment. After the MTT reagent was removed, 160 μL of DMSO was supplemented and shaken for a certain period of time, and its absorbance was detected at 490 nm via an enzyme-labeled instrument.

#### 2.2.11. Transwell invasion assay

Invasion capability of MCF-7 cells and BCSCs was inspected by the transwell chamber with 8 μm pore size, which was performed in a 24-well cell culture plate (Li et al., 2018; Wu et al., 2019). In short, 50 μL of diluted matrigel (matrigel: serum-free MEM medium = 1:12) was added into the Transwell chambers. About 4.5 × 10^5^ MCF-7 cells (3 × 10^5^ breast cancer stem cells) in 200 μL of serum-free MEM medium containing different drugs (free Cur, free ICA, free ICA&Cur, Cur-loaded polymeric micelles, ICA-loaded polymeric micelles, ICA&Cur-loaded polymeric micelles) were afterward inoculated in Matrigel-coated Transwell chamber. Six hundred and fifty microlitres of MEM medium containing 10% FBS was supplemented to bottom layer as attractant. After culturing the two cells for 30 h, the matrigel and un-invasive cells on the inside of the Transwell chamber were cleared away with a cotton swab. The invading cells that adhered to the undersurface of the transwell polycarbonate membrane were fixed with 4% Paraformaldehyde for 30 min, dyed with 670 μL crystal violet for 25 min, and washed with PBS. Eventually, the invasive capability of MCF-7 cells and BCSCs was determined by calculating the cells on the underlying surface of polycarbonate membrane (random selection of 6 visual fields) via an inverted fluorescence microscope.

#### 2.2.12. *In vivo* tumor targeting and distribution

In this study, female nude mice were selected as an animal model and in compliance with animal protection protocols. An appropriate amount of MCF-7 cells suspension was injected into the right anterior side of the back of the nude mouse when the mice weighed approximately 20 g. About 0.2 mL of free DiR, DiR-loaded oHA-FA polymeric micelles and DiR-loaded “nano-actiniaes” (DiR, 0.5 mg/mL) were separately injected into the mice through the tail vein after the tumor volume reached approximately 400 mm^3^. The nude mice were anesthetized with chloral hydrate at different time intervals after injecting free DiR, DiR-loaded oHA-FA polymeric micelles and DiR-loaded “nano-actiniaes”, and images were obtained by small animal live imager. After injection with DiR preparations for 24 h, the tumor-bearing mice were killed to acquire tumor and organs and compared their fluorescence intensity.

#### 2.2.13. *In vivo* evaluation of anti-tumor effect

The mice carrying MCF-7-tumor were used in the research to evaluate the anti-tumor effect of the “nano-actiniaes” *in vivo*. Above exceeding the tumor volume of 200 mm^3^, the nude mice were randomly grouped in five parts (three mice in each group). Five parts of mice were injected intraperitoneally with 200 μL of preparation as follows: saline, free ICA&Cur, Cur-loaded polymeric micelles, ICA-loaded polymeric micelles, and ICA&Cur-loaded polymeric micelles. The bodyweight of the nude mice and tumor volume were measured during the injection of the preparations. After the end of administration, the nude mice were sacrificed and tumor tissues were acquired. The tumor size was obtained via following formula:

$$V\;\left(\text{tumor}\;\text{volume}\right)=\left(\text{length}\times {\text{width}}^{2}\right)\times 0.5$$

#### 2.2.14. Statistical analysis

All experiments were presented as mean ± standard deviation (SD) and performed at least three times. Student’s *t*-test was used for significant comparison and considered *p* < .05 to be statistically significant.

## 3. Results and discussion

### 3.1. Characterization of carriers of Bio-oHA-Hyd-FA

**Figure 3.**
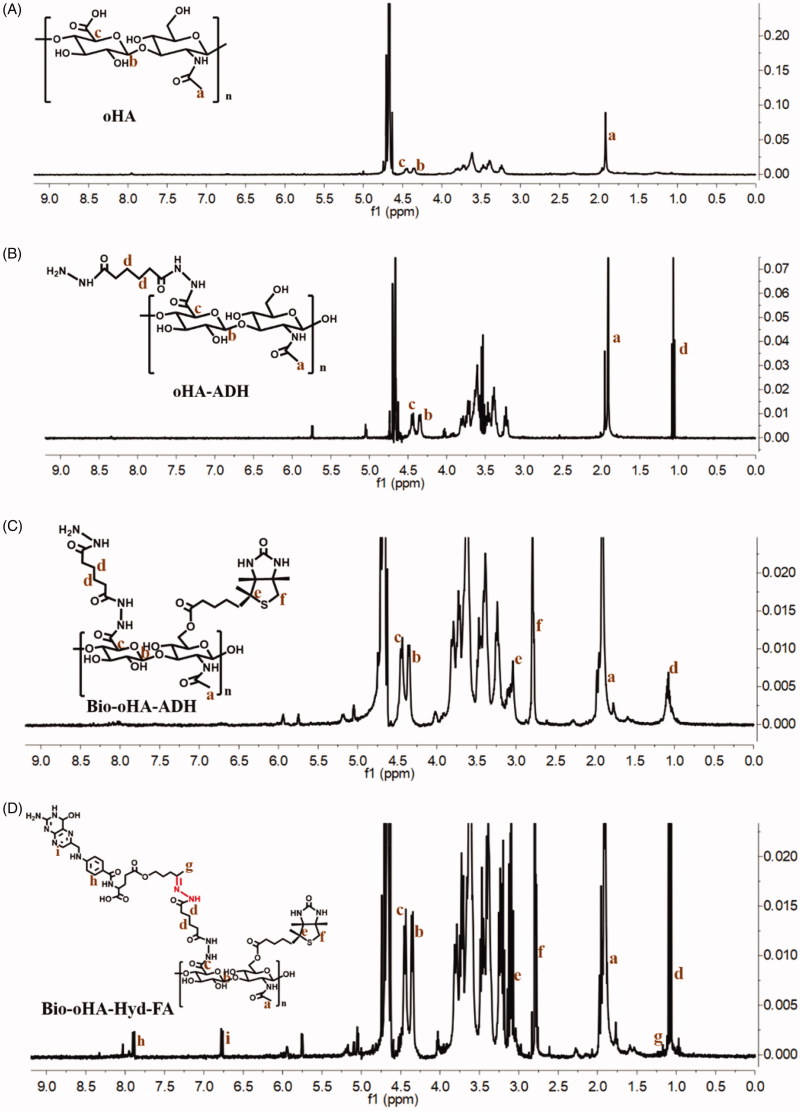
The ^1^H-NMR spectra (Proton nuclear magnetic resonance) of oHA-ADH, Bio-oHA-ADH and Bio-oHA-Hyd-FA.

The synthetic route for the carrier material Bio-oHA-Hyd-FA is shown in Figure 2. The chemical structures of oHA-ADH, oHA-Hyd-FA, Bio-oHA-Hyd-FA were confirmed, as demonstrated in Figure 3. For ^1^H-NMR detection of Bio-oHA-Hyd-FA, the typical signal peak for hyaluronic acid was noticed at 1.948 ppm (a, –CH_3_, methyl group on N-acetylglucosamine). Characteristic peaks at 4.343 ppm (b), and 4.455 ppm (c) were attributed to the hydrogen from D-glucuronic acid structure in hyaluronic acid. The proton signal peaks for ADH (adipic dihydrazide) at 1.127 ppm (d, –CH_2_–CH_2_–) was also clearly observed. The signal peaks for biotin apparently appeared at 2.752 ppm (f, –CH–S) and 3.091 ppm (e, –CH_2_–S–), which proved that biotin was successfully linked. The proton signal peak at 1.21 ppm (g) was assigned to acetylpropyl alcohol methyl group. The chemical shift at 6.786 ppm (h) was assigned to hydrogen from benzene ring of folic acid. The peak located at 7.82 ppm (i) was ascribed to hydrogen on the pyrazine ring from folic acid. These spectra outcomes proved amphiphilic drug carriers Bio-oHA-Hyd-FA were successfully synthesized.

### 3.2. Characterization of ICA&Cur “nano-actiniaes”

**Figure 4.**
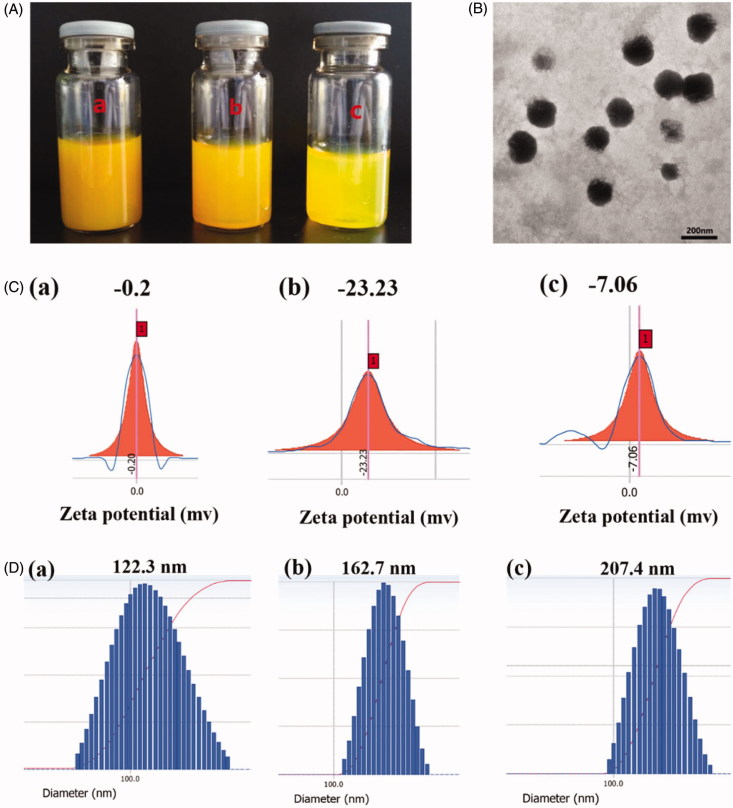
(A) Photograph of Bio-oHA-Hyd-FA micelles solution was filtrated through Millipore membrane of 800 nm (a), 450 nm (b) and 220 nm (c). (B) The TEM images of the “nano-actiniaes”. (C) The zeta potential of the “nano-actiniaes”. (D) The size distribution of the “nano-actiniaes”.

Preparation of ICA&Cur “nano-actiniaes” by self-assembly in water solution using Bio-oHA-Hyd-FA conjugates is shown in Figure 4. As indicated in Table 1, the particle diameter, EE%, DL%, PDI and zeta potential for the polymeric micelles were detected. The ICA&Cur “nano-actiniaes” were smaller and uniformly dispersed with a particle size of 162.7 ± 5 nm. TEM images showed that the ICA&Cur “nano-actiniaes” had spherical morphology. For the study, zeta potential for the ICA&Cur “nano-actiniaes” was −23.23 ± 2.6 mv. Moreover, the zeta potential for nano-micelles exceeded −20 mv, indicating the comparatively stable micelle system. In addition, the PDI for the ICA&Cur “nano-actiniaes” was 0.027 less than 0.2, illustrating the micelles’ uniform dispersion.

**Table 1.** The physiochemical properties of “nano-actiniaes”.

Ratio of medication (mg)	Size (nm)	ζ Zeta (mV)	PDI	EE%		DL%	
Icariin: curcumin				Icariin	Curcumin	Icariin	Curcumin
0.5:0.5	122.3 ± 11	−0.7 ± 0.2	0.224 ± 0.03	33.33 ± 1.7	45.93 ± 2.4	2.78 ± 0.8	3.83 ± 1.1
1.5:0.5	167.5 ± 5	−23.23 ± 2.6	0.027 ± 0.01	40.78 ± 1.3	56.49 ± 1.6	3.40 ± 1.4	5.04 ± 2.1
1.8:1.2	207.4 ± 8	−7.06 ± 2.3	0.089 ± 0.05	34.23 ± 3.5	54.91 ± 4.3	2.34 ± 0.6	4.58 ± 1.3

### 3.3. *In vitro* pH-responsive ICA&Cur release research

**Figure 5.**
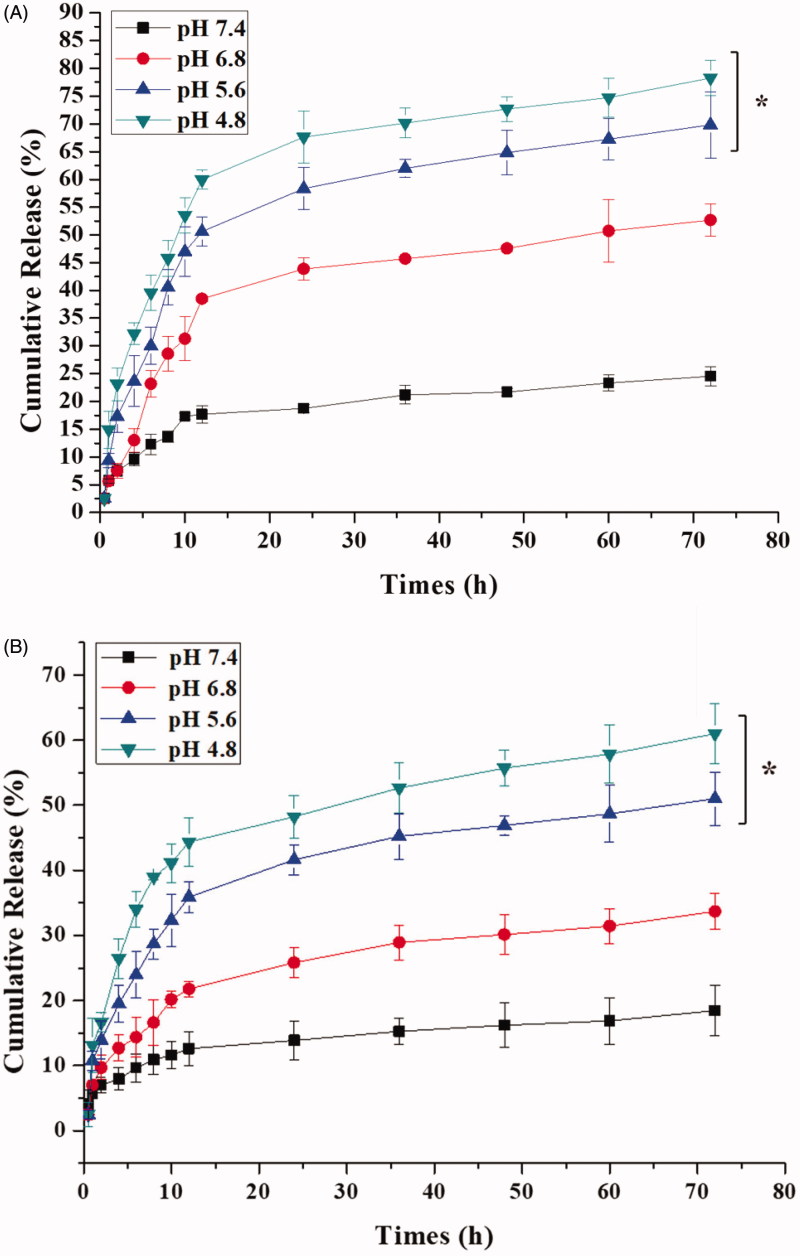
(A) *In vitro* release profiles of Cur from the “nano-actiniaes” at different pH condition. (B) *In vitro* release profiles of ICA from the “nano-actiniaes” at different pH condition. (*indicates *p* < .05).

The pH-responsive capability of ICA&Cur “nano-actiniaes” was researched in different conditions, as displayed in Figure 5. The cumulative release of Cur and ICA increased with decreasing pH values, which indicated that the pH-sensitive groups in the nano-carriers were destroyed in an acidic environment, making anticancer drugs easier to release. This affirmed that the ICA&Cur “nano-actiniaes” were more likely to release anticancer drugs under acidic condition, and the drug carrier had a pH-sensitive property *in vitro*.

### 3.4. Cellular uptake and localization researches

As demonstrated in Figure 6, the green signal was discovered in MCF-7 cells and BCSCs due to fluorescent labeling of curcumin. As can be seen from the results in Figure 6(A), the optimal uptake time for the Bio-oHA-Hyd-FA micelles was about 3–4 h. This indicated that the uptake efficiency of the micelles by MCF-7 cell lines and BCSCs was affected by time. Moreover, the fluorescent signal from the micelles was stronger, probably due to folic acid, biotin and CD44 targeting of the Bio-oHA-Hyd-FA carriers. The uptake results in Figure 6(B) revealed that the green fluorescence signal for the tumor cells was stronger at a drug concentration of 20 μg/mL. This suggested that the uptake efficiency by the cancer cells was also affected by drug concentration. In addition, due to the pH-sensitivity property of the ICA&Cur “nano-actiniaes”, its green fluorescence was stronger than other groups.

Figure 6.(A, B) The MCF-7 and BCSCs cell uptake of free Cur, free ICA&Cur, Cur-loaded micelles and ICA&Cur-loaded micelles. (C (a, b)) Internalization imaging of Bio-oHA-Hyd-FA in MCF-7 cells and BCSCs after 4 h incubation. The cells were stained with Hoechst 33342 and the nucleus showed blue fluorescence.

**Figure:**
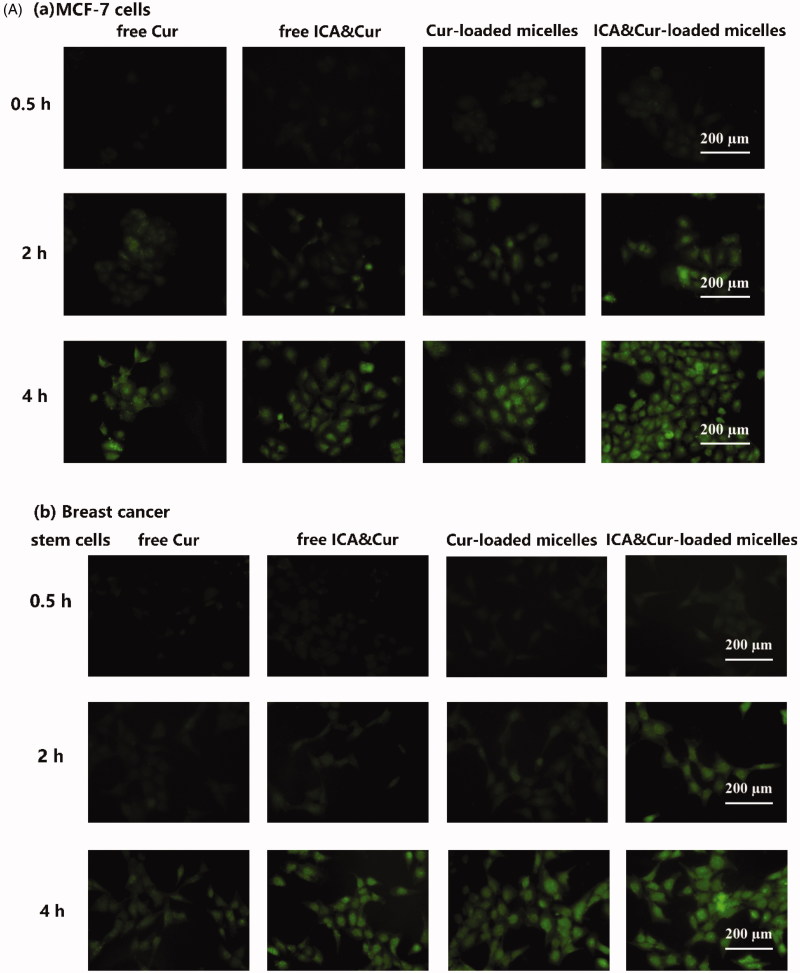

**Figure:**
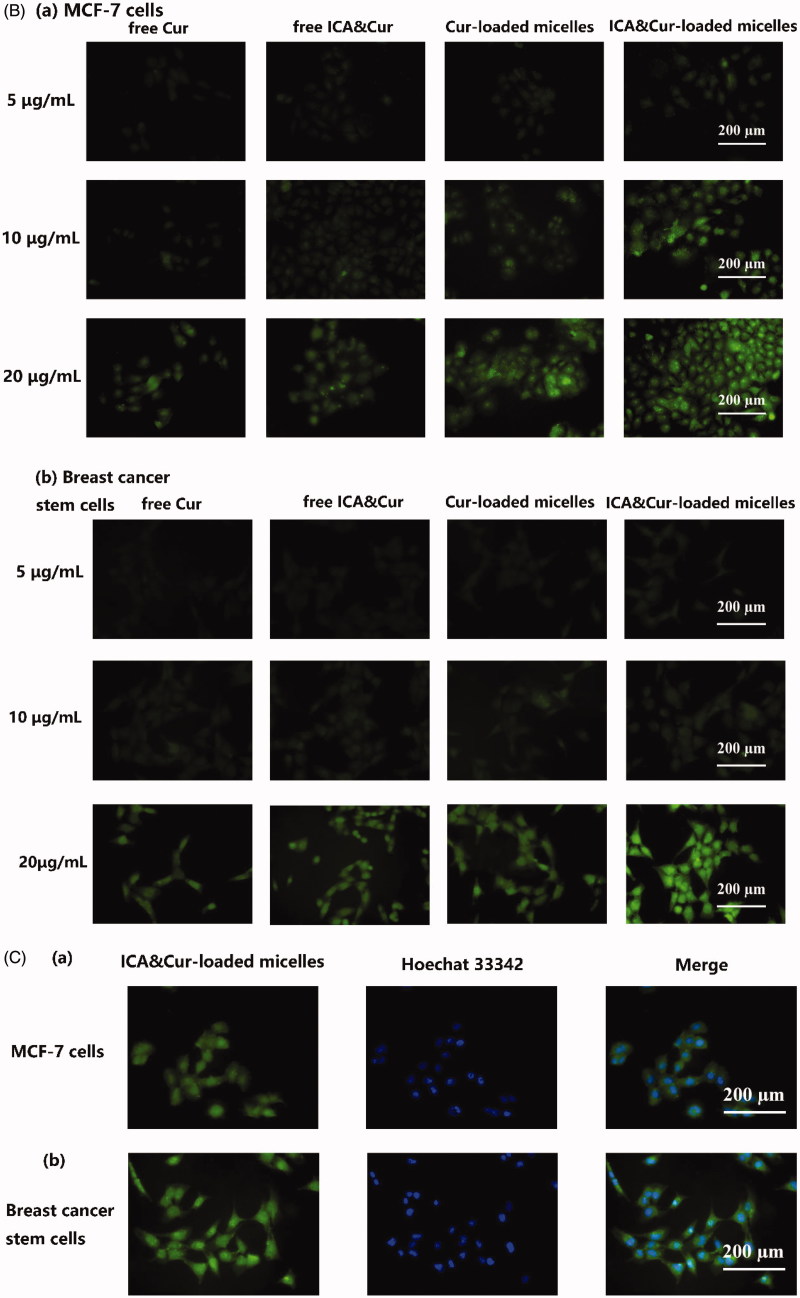

As displayed in Figure 6(C), the green fluorescent signal was mainly concentrated in the cytoplasm after staining with Hoechst 33342 (nuclear stained blue). The above outcomes demonstrated that the “nano-actiniaes” effectively targeted cancer cells and produced anti-tumor toxicity.

### 3.5. Cell cytotoxicity of Bio-oHA-Hyd-FA and ICA&Cur “nano-actiniaes”

This study evaluated the cytotoxicity of Bio-oHA-Hyd-FA material, free Cur, free ICA, free ICA&Cur, Cur-loaded polymeric micelles, ICA-loaded polymeric micelles, and ICA&Cur-loaded polymeric micelles in MCF-7 cells and BCSCs via MTT reagent. As displayed in Figure 7(A), there was no obvious difference in the effect of Bio-oHA-Hyd-FA materials on cell viability of MCF-7 cells and BCSCs at 48 h. When the Bio-oHA-Hyd-FA materials concentration reached 500 μg/mL, the survival ratio for BCSCs and MCF-7 cells was still higher than 75%, demonstrating low toxicity and ideal safety of Bio-oHA-Hyd-FA. As illustrated in Figure 7(B–E), the results from the MTT assay demonstrated that the ICA&Cur-loaded polymeric micelles group had significantly higher cytotoxicity compared to the free Cur group, free ICA group, free ICA&Cur group, Cur-loaded polymeric micelles group and ICA-loaded polymeric micelles group, probably because combination of icariin and curcumin had stronger inhibitory effect on cancer cells.

Figure 7.(A) The cytotoxicity of Bio-oHA-Hyd-FA carrier in MCF-7 cells and BCSCs. (B (a, b)) The cell viabilities of free Cur, free ICA and free ICA&Cur at 24 h and 48 h in MCF-7 cells, respectively. (C (a, b)) The cell viabilities of Cur-loaded Bio-oHA-Hyd-FA micelles, ICA-loaded Bio-oHA-Hyd-FA micelles and ICA&Cur-loaded Bio-oHA-Hyd-FA micelles at 24 h and 48 h in MCF-7 cells, respectively. (D (a, b)) The cell viabilities of free Cur, free ICA and free ICA&Cur at 24 h and 48 h in BCSCs, respectively. (E (a, b)) The cell viabilities of Cur-loaded Bio-oHA-Hyd-FA micelles, ICA-loaded Bio-oHA-Hyd-FA micelles and ICA&Cur-loaded Bio-oHA-Hyd-FA micelles at 24 h and 48 h in BCSCs, respectively. (*indicates *p* < .05).

**Figure:**
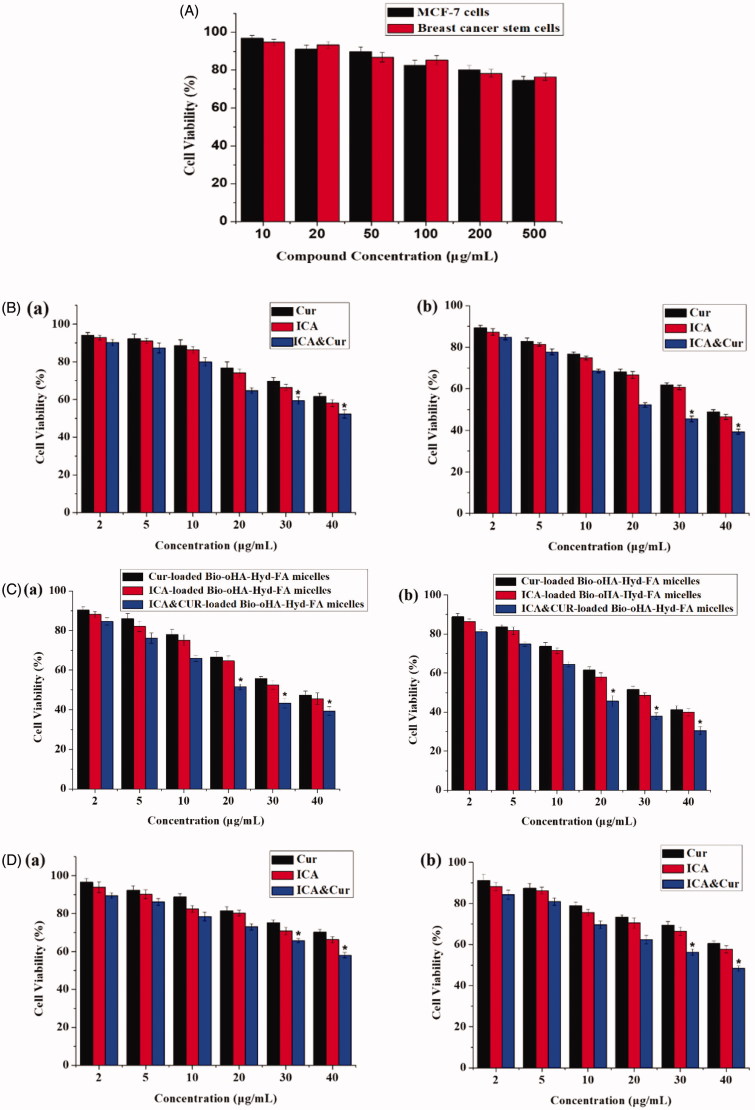

**Figure:**
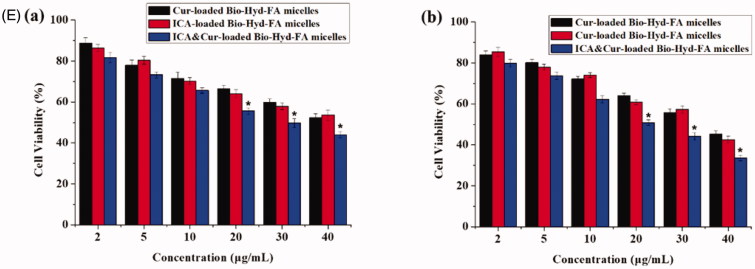

### 3.6. Transwell invasion assay

**Figure 8.**
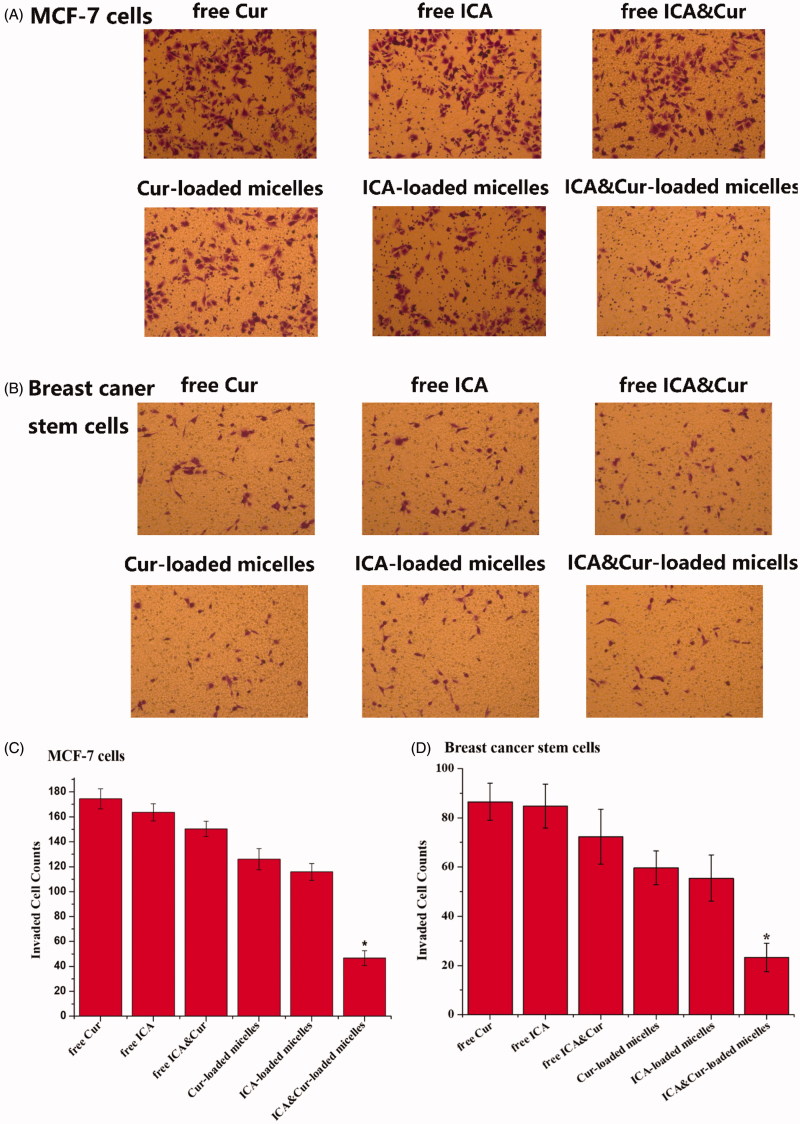
The influence of Bio-oHA-Hyd-FA on invasion ability of MCF-7 cells and BCSCs. (A, B) Images describe invaded MCF-7 cells and BCSCs on the undersurface of the transwell polycbonate membrane in different control groups. (C, D) The invasion numbers of corresponding A and B. (*indicates *p* < .05).

As displayed in Figure 8, MCF-7 cells and BCSCs invasion was detected by transwell invasion assays. Results from the invasion experiment illustrated that the number of invading cells was significantly reduced after adding ICA&Cur “nano-actiniaes”. The ICA&Cur “nano-actiniaes” obviously inhibited tumor cell invasion. This demonstrated that the combined delivery of curcumin and icariin made an important impact in MCF-7 cells and BCSCs invasion.

### 3.7. *In vivo* tumor targeting and distribution

**Figure 9.**
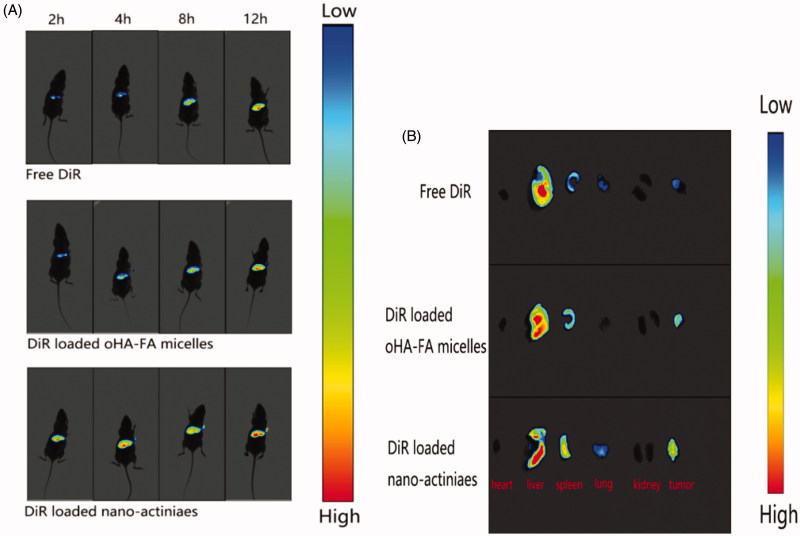
(A) In vivo fluorescence intensity imaging of the MCF-7 tumor bearing nude mice tail vein injection with different DiR preparations at 2 h, 4 h, 8 h, and 12 h. (B) The fluorescent image of organ tissue obtained from the nude mice 24 h after administration.

In order to in-depth assess the anti-tumor effect of “nano-actiniaes”, real-time imaging system was used to detect the distribution and targeting of different DiR preparations *in vivo*. As displayed in Figure 9(A), compared to DiR-loaded oHA-FA polymeric micelles and free DiR, the DiR-loaded “nano-actiniaes” realized significant accumulation in tumor with passage of time. Moreover, there was almost no fluorescence aggregation at the tumor site in the free DiR group, which demonstrated that free DiR has no targeting effect on tumor tissue. Twelve hours after DiR-loaded “nano-actiniaes” injection, the tumor site had stronger fluorescent signal, which indicated that the “nano-actiniaes” ameliorated tumor targeting of anti-cancer drugs. In addition, the fluorescence from the DiR-loaded “nano-actiniaes” was significantly stronger than that of the other control groups in the isolated tumor tissues, revealing that oHA, FA, and biotin made the anticancer drugs have better targeting properties, as illustrated in Figure 9(B). Consequently, these results evidenced that the “nano-actiniaes” effectively delivered anticancer drugs to tumor tissues.

### 3.8. *In vivo* evaluation of anti-tumor effect

**Figure 10.**
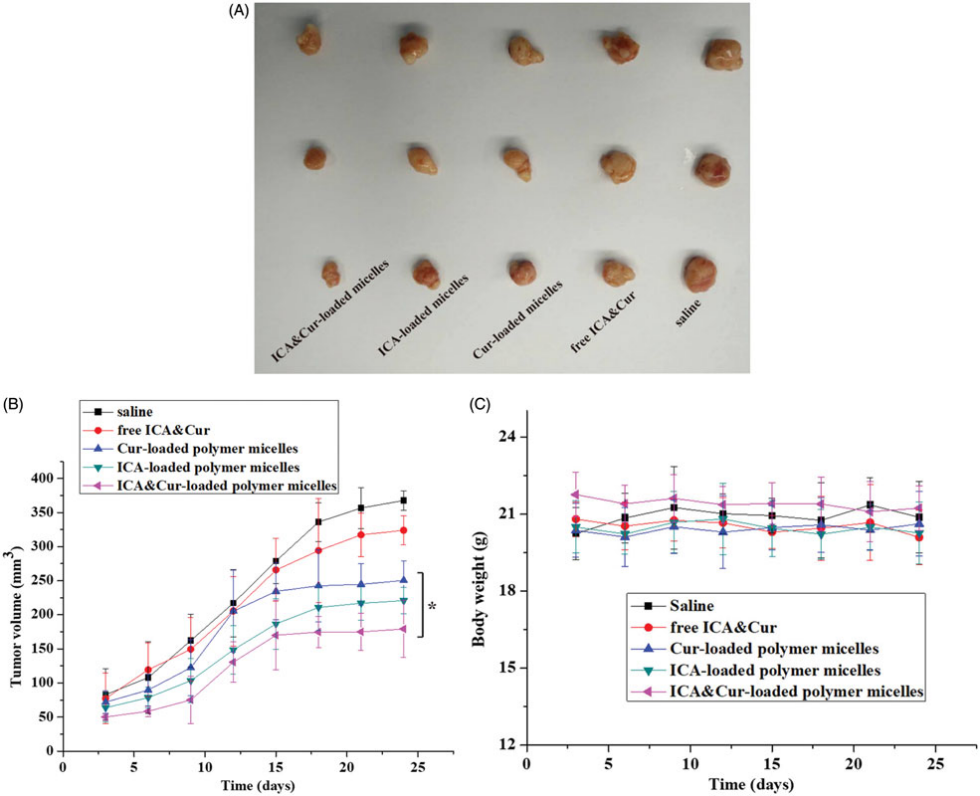
(A) The picture of isolated tumor tissue. (B, C) Tumor volume and body weight changes of nude mice during administration. (*indicates *p* < .05).

As displayed in Figure 10(A), the tumor size of the ICA&Cur “nano-actiniaes” was obviously smaller than that of the other control groups, which demonstrated the development of tumors inhibited by ICA&Cur “nano-actiniaes”. The tumor volume of the saline group was the largest. The tumor size of free ICA&Cur exhibited a slight anti-tumor efficacy. As illustrated in Figure 10(B,C), the tumor volume of the nude mice injected with the ICA&Cur “nano-actiniaes” group was the smallest, and there was no significant difference in the bodyweight of the nude mice, indicating that the micelles had reliable safety. The above results revealed that the ICA&Cur “nano-actiniaes” possessed obviously antitumor efficiency. Importantly, these results also confirmed that curcumin and icariin synergistically inhibit tumor growth.

## 4. Conclusion

In this research, CD44-targeted, biotin-targeted, and folate-targeted amphiphilic Bio-oHA-Hyd-FA drug carriers were successfully synthesized for delivery of ICA&Cur to tumor cells and cancer stem cell. After elementary evaluation, the proton nuclear magnetic resonance (^1^H NMR spectrogram) for the conjugates demonstrated that the Bio-oHA-Hyd-FA carriers had a pH-sensitive hydrazone bond group that made it pH-sensitive. The Bio-oHA-Hyd-FA carriers encapsulated the anticancer drugs ICA and Cur by self-assembly into nanomicelles (like “nano-actiniaes”) and simultaneously delivered the two drugs to the tumor cells. The encapsulation rates of curcumin and icariin were 56.49% and 40.78%, respectively, and the drug loading was separately 5.04% and 3.4%. Furthermore, cell uptake experiments demonstrated that the Bio-oHA-Hyd-FA carriers accurately targeted cancer cells. Moreover, cell cytotoxicity study revealed that the ICA&Cur “nano-actiniaes” had low toxicity and reliable safety in tumor cells and effectively boosted cancer cell apoptosis. In addition, the transwell invasion assay showed that the ICA&Cur “nano-actiniaes” effectively inhibited tumor cell invasion. Ultimately, *in vivo* tumor inhibition research proved that the ICA&Cur “nano-actiniaes” displayed stronger anti-tumor effect. The above outcomes demonstrated that the Bio-oHA-Hyd-FA drug carriers, with pH-sensitive property and multiple targets, possessed reliable prospect for targeting cancer cells and cancer stem cells, and will achieve combination therapy of two anticancer drugs to reduce drug resistance.
